# Does BCG vaccination protect against infection with *M. tuberculosis*?

**DOI:** 10.5588/ijtld.21.0607

**Published:** 2022-06-01

**Authors:** P. T. Pelzer, Y. Smit, E.W. Tiemersma, N. T. Huong, N. V. Nhung, F. Cobelens

**Affiliations:** 1 KNCV Tuberculosis foundation, Technical division, The Hague, The Netherlands; 2 Department of Global Health and Amsterdam Institute for Global Health and Development, Amsterdam University Medical Centers, Amsterdam, The Netherlands; 3 NTP Vietnam, National Lung Hospital, Hanoi, Viet Nam; 4 KNCV Tuberculosis Foundation, Country office in Vietnam, Hanoi, Viet Nam

**Keywords:** tuberculosis, infection, BCG, tuberculin skin test, vaccine efficacy

## Abstract

**BACKGROUND:**

Protection against infection by the bacille Calmette-Guérin vaccine against *Mycobacterium tuberculosis* remains a subject of controversy. We investigated the association between BCG vaccination at birth and infection by *M. tuberculosis*.

**MATERIAL AND METHODS:**

This was a secondary analysis of data from tuberculin skin test (TST) surveys in Vietnamese schoolchildren between 1988 and 2001. We investigated whether a BCG scar was associated with a lower prevalence of TST positivity, adjusting for BCG-induced variation by varying cut-off values for a positive TST.

**RESULTS:**

We found a positive association between BCG scar and TST positivity. The strength of the association decreased with increasing TST cut-off values; however, it never inverted significantly, irrespective of geographic region and survey year.

**CONCLUSION:**

In Vietnam, BCG vaccination was not associated with reduced *M. tuberculosis* infection prevalence as measured using TST. This in contrary to a similar study conducted in Tanzania. These contradictory findings may be explained by geographical differences and the relatively high prevalence in Vietnam of the *M. tuberculosis* Beijing genotype, which is reported to be capable of circumventing BCG-induced immunity.

## Abstract (fr)

**CONTEXTE:**

La protection conférée par le vaccin par le bacille de Calmette-Guérin (BCG) contre l’infection à *Mycobacterium tuberculosis* reste un sujet controversé. Nous avons analysé l’association entre la vaccination par le BCG à la naissance et l’infection à *M. tuberculosis*.

**MATÉRIEL et MÉTHODES:**

Il s’agissait d’une analyse secondaire des données d’enquêtes ayant réalisé des tests cutanés à la tuberculine (TST) chez des écoliers vietnamiens entre 1988 et 2001. Nous avons cherché à savoir si une cicatrice laissée par le vaccin par le BCG était associée à une plus faible prévalence de résultats positifs au TST, en ajustant pour la variation induite par le vaccin selon diverses valeurs seuils pour un TST positif.

**RÉSULTATS:**

Nous avons observé une association positive entre une cicatrice BCG et un résultat positif au TST. La force de l’association diminuait à mesure que les valeurs seuils du TST augmentaient, mais sans jamais s’inverser de manière significative, indépendamment de la zone géographique et de l’année d’étude.

**CONCLUSION:**

Au Vietnam, la vaccination par le BCG n’a pas été associée à une moindre prévalence de l’infection à *M. tuberculosis*, telle que mesurée par TST et contrairement à une étude similaire réalisée en Tanzanie. Ces résultats contradictoires peuvent s’expliquer par des différences géographiques et par la prévalence relativement élevée au Vietnam du génotype Beijing de *M. tuberculosis*, qui serait capable de contourner l’immunité induite par la vaccination par le BCG.

TB is one of the largest global causes of mortality due a single infectious agent, only recently surpassed by COVID-19, with 1.5 million TB deaths in 2019.^1^ Infection by *Mycobacterium tuberculosis* is the precursor to TB disease. Approximately 5% of those infected will develop TB disease. In 2016, 23% of the world’s population was estimated to be infected with *M. tuberculosis*.^2^ While there are 14 vaccines in the pipeline, the bacille Calmette-Guérin (BCG) remains the only vaccine recommended by the WHO. The BCG vaccine has been shown to prevent TB disease (particularly TB meningitis and miliary TB) in young children.^3^ While BCG was previously thought to protect mainly against disease, several studies have indicated that BCG might in addition provide protection against TB infection.^4^ However, since infection with *M. tuberculosis* cannot be measured with certainty, this remains controversial. A meta-analysis of a small number of observational studies among children found a reduced risk for interferon-gamma release assay (IGRA) conversion among BCG-vaccinated compared to non-vaccinated children.^5^ The BCG protection against infection efficacy was 27% (risk ratio 0.73, 95% confidence interval [CI] 0.61–0.87), but sub-analyses showed protection against infection only in countries at latitudes more than 40 degrees from the equator.^5^

Among Tanzanian school children, large tuberculin skin test (TST) induration sizes were seen less often in BCG vaccinated than in non-vaccinated children. This suggests protection against infection by *M. tuberculosis* from BCG,^6^ since large TST induration sizes compared to small sizes are more likely to reflect *M. tuberculosis* infection rather than earlier BCG vaccination or exposure to non-tuberculous mycobacteria (NTM).^7^ This association was not seen in an Indonesian cohort using an incidence endpoint.^8^ These opposite findings might be ascribed to geographical differences and the relatively high prevalence of the *M. tuberculosis* Beijing genotype in Indonesia, which is reported to be capable of circumventing BCG-induced immunity.^9^

We investigated whether a similar association between BCG vaccination and *M. tuberculosis* infection observed in Tanzania exists in the South-East Asian context using similar methods by quantifying the association between BCG scar and positive TST among Vietnamese school children using differential cut-off values for a positive test.

## METHODS

### Study population

This was a secondary analysis of consecutive tuberculin surveys conducted in Vietnam between 1988 and 2001, originally done to estimate the annual risk of TB infection (ARTI) in the country. Further details of the surveys have been published elsewhere.^10^ The surveys were conducted in the following provinces: Hanoi (1988, 1993, 1999), Hai Phong (1990, 1996, 2001), Thua Thien-Hué (TTH) (1991, 1997), Quang Nam-Da Nang (1994, 2000), Ho Chi Minh City (HCMC) (1991, 1993, 1997) and Dong Thap (1990, 1995, 2000). In total, 137 schools were selected randomly proportional to district size from all districts within the province. All children enrolled in grade 1 and grade 2 of the selected schools were listed with their age and sex. In Vietnam, the BCG policy, introduced in 1985, involves immunisation at birth.^11^

### Survey procedures

The Mantoux TST was performed by trained health survey teams as per international guidelines,^12^ using 2 tuberculin units (except in Hanoi in 1988 and HCMC in 1993, where 1 unit was used) of purified protein derivative RT-23 with Tween (Statens Serum Institute, Copenhagen, Denmark) administered with 1 ml disposable syringes and disposable 26-gauge needles on the dorsal side of the left forearm. The presence of the typical scar that the BCG vaccine usually causes was checked and recorded by the person administering the TST.^10^ TST reactions were read after 48–72 hours.

### Analysis

A total of 124,348 schoolchildren were included in the survey. Exclusion criteria for the purpose of our analyses were age <5or >14 years, and/or having missing data for either age, BCG status, TST reaction size (induration) or sex.

Analyses were conducted using Stata v15.1 (Stata Corp, College Station, TX, USA). We used a method published earlier by taking into account differences in TST reaction sizes to avoid cross-reactivity.^6^ In brief, we conducted univariable and multivariable logistic regression to calculate crude and adjusted odds ratios (ORs) for a positive TST in children with a BCG scar vs. those without a BCG scar for each pre-determined cut-off value of induration size, increasing this cut-off from 10 mm to 26 mm. This method is based on earlier observations that the distributions of TST reaction sizes due to BCG vaccination or sensitisation to NTM have a lower mean than those due to *M. tuberculosis* infection.^13^–^15^ Therefore, the higher the cut-off value for declaring a TST result positive, the higher its specificity for *M. tuberculosis* infection, and the closer the observed OR for the association between BCG vaccination status and *M. tuberculosis* infection will be to the true value.^16^,^17^

The analyses were stratified a priori for survey. We also assessed the need for stratification by age and sex. All multivariable models were adjusted for age, sex and year. All *P* values and 95% CIs were adjusted for clustering by province and school by calculating robust standard errors using Stata’s *svy* command. We considered *P* < 0.05 as statistically significant.

### Ethics statement

As this was a secondary analysis of existing data, no ethical approval was required.

## RESULTS

From the 124,348 children enrolled in the initial survey, we excluded 83 children aged <5or >14 years, or having one or more missing values (*n* = 3,306), leaving 120,959 children for our analysis (Supplementary Data 1). There were no notable differences between included and excluded children (data not shown). The overall proportion of children with a BCG scar was 48% (Table 1). This varied per survey, ranging from 9.6% in Hanoi in 1988 to 83.7% in HCMC in 1993. Boys and girls were equally distributed among those with and without a BCG scar.

**Table 1:** Characteristics of Vietnamese school children by BCG scar status

	Total *n*	BCG scar		No BCG scar	
	
		*n*	%	*n*	%
Girls	57,381	27,622	48.1	29,759	51.9
Boys	63,578	31,572	49.7	32,006	50.3
Age, years, median [IQR]	7 [6–7]	7 [6–8]		7 [6–8]	
Survey					
Danang-Quangnam 1994	9,808	4,063	41.4	5,745	58.6
Danang-Quangnam 2000	10,538	8,423	79.9	2,115	20.1
Dong Thap 1990	5,092	548	10.8	4,544	89.2
Dong Thap 1995	3,942	1,718	43.6	2,224	56.4
Dong Thap 2000	3,280	2,742	83.6	538	16.4
Hai Phong 1990	7,509	1,498	19.9	6,011	80.1
Hai Phong 1996	8,244	5,164	62.6	3,080	37.4
Hai Phong 2001	7,039	5,756	81.8	1,283	18.2
Hanoi 1988	10,800	1,036	9.6	9,764	90.4
Hanoi 1993	9,481	2,022	21.3	7,459	78.7
Hanoi 1999	9,374	7,505	80.1	1,869	19.9
HCMC districts 1991	8,944	3,971	44.4	4,973	55.6
HCMC districts 1993	4,648	3,891	83.7	757	16.3
HCMC districts 1997	7,876	6,009	76.3	1,867	23.7
Thua Thien-Hué 1991	7,145	1,056	14.8	6,089	85.2
Thua Thien-Hué 1997	7,239	3,792	52.4	3,447	47.6
Total	120,959	59,194	48.9	61,765	51.1

BCG = bacille Calmette-Guérin; IQR = interquartile range; HCMC = Ho Chi Minh City.

**Figure 1:**
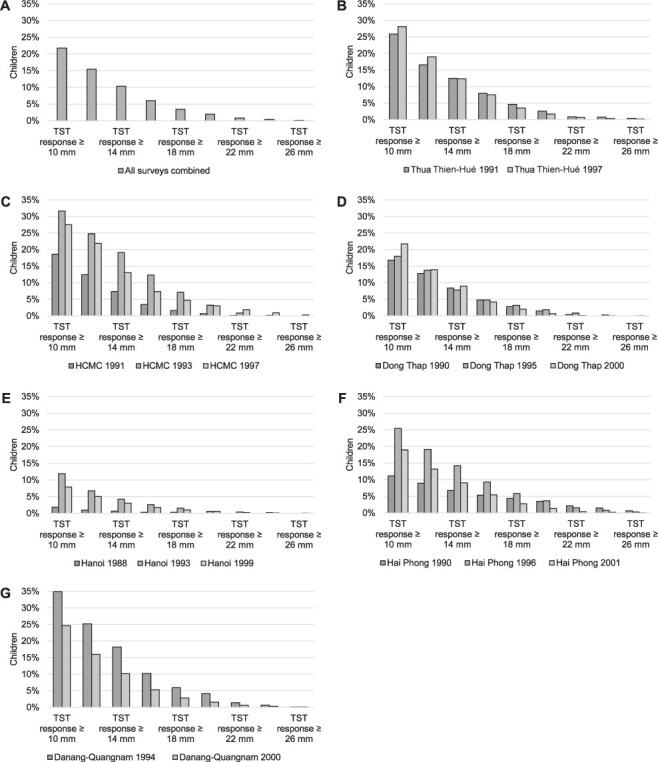
Proportion of children with with positive TST at different cut-off points, among children with a BCG scar. A) All surveys combined; B) Thua Thien-Hué (1991, 1997); C) HCMC districts (1991, 1993, 1997); D) Dong Thap (1990, 1995, 2000); E) Hanoi (1988, 1993, 1999); F) Hai Phong (1990, 1996, 2001); G) Danang-Quangnam (1994, 2000). TST = tuberculin skin test; HCMC = Ho Chi Minh City; BCG = bacille Calmette-Guérin.

The proportion of children with BCG scar increased from 10% in 1988 to 82% in 2001 (Supplementary Data 2). The distribution of the children’s induration sizes differed by survey, although a similar trend was seen throughout. Induration sizes that were multiples of five were reported more frequently, suggesting digit preference (Supplementary Data 3). The induration size distribution in Hanoi in 1988 differed substantially from that of other surveys, with almost all children having an induration size ≤5 mm. The distribution of TST positivity by induration size among children with a BCG scar is shown in Figure 1.

**Figure 2:**
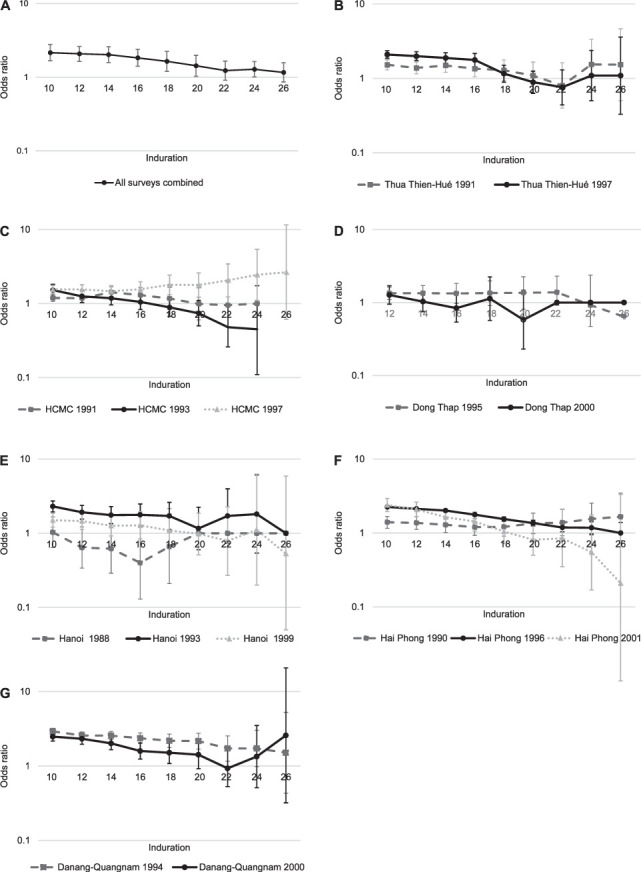
Odds ratios (for positive TST for children with BCG scar vs. no-BCG scar for each respective cut-off value, adjusted for age, sex and survey year) with 95% CIs combined and per province and survey year. Graphs presented on logarithmic scale; error bars display 95% CI; all 95% CIs correct for cluster sampling. A) All surveys combined; B) Thua Thien-Hué (1991, 1997); C) HCMC districts (1991, 1993, 1997); D) Dong Thap (1990, 1995, 2000); E) Hanoi (1988, 1993, 1999); F) Hai Phong (1990, 1996, 2001); G) Danang-Quangnam (1994, 2000). HCMC = Ho Chi Minh City; CI = confidence interval.

Table 2 shows the crude and adjusted ORs for each induration cut-off point ranging from 10 mm to 26 mm. The frequency and percentage of positive tests based on induration cut-off value among children with and those without BCG scar is shown in Supplementary Data 4. The OR for a positive TST for the individuals with a BCG scar vs. those without a BCG scar was greater than 1 at 10- and 12-mm cutoff points (Table 2). The OR gradually decreased, with increasing cut-off value for a positive TST to approximately 1 in the 26 mm cut-off category. The age-, sex-, and survey year-adjusted ORs for the association between BCG scar and TST positivity followed a similar trend, at slightly higher values as the unadjusted ORs, although these never dropped below 1 (Figure 2, Panel A). There was no effect modification by age or sex (Supplementary Data 5).

**Table 2:** Crude and adjusted odds ratios between presence of BCG vaccination scar and TST result, at different cut-off points * ^†^

Induration size	Unadjusted OR (95% CI)	Adjusted OR^‡^ (95% CI)
TST response cut-off ≥10 mm	2.14 (1.46–3.12)	2.15 (1.67–2.78)
TST response cut-off ≥12 mm	2.02 (1.44–2.84)	2.08 (1.64–2.62)
TST response cut-off ≥14 mm	1.92 (1.36–2.7)	2.02 (1.58–2.59)
TST response cut-off ≥16 mm	1.71 (1.19–2.46)	1.83 (1.41–2.38)
TST response cut-off ≥18 mm	1.49 (0.98–2.27)	1.64 (1.2–2.24)
TST response cut-off ≥20 mm	1.32 (0.83–2.11)	1.43 (1.03–1.98)
TST response cut-off ≥22 mm	1.16 (0.64–2.1)	1.23 (0.91–1.65)
TST response cut-off ≥24 mm	1.14 (0.64–2.04)	1.28 (1.01–1.63)
TST response cut-off ≥26 mm	0.97 (0.5–1.88)	1.16 (0.86–1.57)

* OR for positive TST for children with BCG scar vs. no-BCG scar for each respective cut-off value.

† 95% CIs adjusted for design effect due to stratification (regions) and cluster sampling (provinces and schools).

‡ Adjusted for age, sex and survey year.

BCG = bacille Calmette-Guérin; TST = tuberculin skin test; OR = odds ratio; CI = confidence interval.

Figure 2 shows the ORs for the overall association between BCG and TST positivity along the 10–26 mm range, and separately for the six provinces and the different survey years. At a cut-off value of 10 mm, the adjusted OR for a positive TST in the children with a BCG scar compared to those without such a scar was >1 in all provinces. In Danang-Quangnam, Hai Phong, Hanoi 1993 and 1999, and TTH, the OR decreased with increasing cut-off values. Hanoi 1988 showed an opposite trend compared to the subsequent survey rounds, with an inverse association (BCG scar associated with lower TST positivity) for cut-off values <20 mm (Figure 2, Panel E). HCMC had a divergent association over the survey years (Figure 2, Panel C). HCMC 1991 and 1993, Dong Thap 2000, Hai Phong 2001, and TTH 1991 and 1997 showed inverse associations at the higher cut-off values (18–26 mm), although these were not statistically significant. Hanoi 1988 and 1999 showed inverse associations at 12–18 mm cut-offs. Some survey analyses had highly imprecise estimates at the higher (>24 mm) cut-off values.

## DISCUSSION

In our analysis across most surveys, having a BCG scar was associated with an increased proportion of TST positivity, even at very high cut-off levels for the induration size where the TST is assumed to have highest specificity for *M. tuberculosis* infection. This suggests that in Vietnam, BCG vaccination given at birth did not protect primary school age children against *M. tuberculosis* infection over the period 1988–2003.

Stratified by survey, the association between BCG scar and TST induration size showed a similar decreasing trend up to induration cut-off of 20 mm, apart from two surveys in different provinces (Hanoi 1988 and HCMC 1997). Some surveys did show an inverse association at the higher cut-off values, although this was never statistically significant and had large confidence intervals. Geographic variation in the distribution of NTM could account for some of these differences. NTM exposure could cause false-positive TST results due to cross-reactivity,^18^ the effect of which would depend on the relative prevalence of *M. tuberculosis* infection compared to NTM infection, and thereby also on TB incidence. NTM are also thought to modulate the immunogenicity and efficacy of BCG.^19^ The prevalence of NTM in Vietnam is assumed to be higher in the South than in the North,^20^ as the South has a tropical climate, whereas the North has a winter season. In the United States and Australia, a higher prevalence of NTM-related disease was found in areas with higher temperatures and humidity.^21^,^22^ In addition, there were regional differences in TB incidence in Vietnam at the time of the survey. Annual TB case notification rates decreased between 1990 and 1995 in TTH, HCMC and Dong Thap, increased in Hanoi and Quang Nam-Da Nang,^10^ and remained steady in Hai Phong. Between 1996 and 2003, notifications declined in all provinces except HCMC. The low induration sizes found in 1988 in Hanoi may be related to the very low incidence of TB in that province before 1992, but may also be in part caused by the fact that this survey used 1 tuberculin unit. Such effect was not seen in HCMC in 1993, possibly because the intensity of *M. tuberculosis* infection was much higher there.

The fact that these findings contradict our results published from Tanzania^6^ could be due to several factors, including variability in the protective effect of BCG immunisation against *M. tuberculosis* infection. The suitability of the study method used could be different in the two countries due to NTM distribution. We assumed that the misclassification of the TST endpoint is random, i.e., not associated with BCG status, but that may not be the case if there is uncontrolled confounding. BCG vaccination status as well as NTM exposure may both be associated with socio-economic status,^20^ for which we had no data. Moreover, this is expected to influence TST results at the lower cut-offs, with decreasing effect as the cutoff increases. Second, Vietnam has a relatively high prevalence of TB caused by the Beijing genotype compared to Tanzania (58% vs. <6.5%).^23^,^24^ The Beijing lineage has been shown to be more virulent than other lineages,^9^ and certain Beijing sub-lineages are possibly able to circumvent BCG-induced immunity.^25^ Furthermore, meta-analyses of BCG vaccination trials have suggested that latitude may be a main factor explaining variation in BCG vaccine efficacy,^26^ the protective effect against TB disease being higher in trials that were conducted further from the Equator. However, this does not provide explanation for our contrasting findings: Vietnam lies in the Northern Hemisphere, approximately 500 miles further from the Equator than Tanzania, which is in the Southern Hemisphere. Finally, the protective effect of BCG vaccination might be cancelled out by continuous exposure to *M. tuberculosis* and thus depend on background TB incidence. In 2000, TB incidence in Vietnam was estimated to be 503/100,000 population (95% CI 95–1240), while this was only 296/100,000 population (95% CI 90–624) in Tanzania.^27^

In Vietnam, a BCG vaccine manufactured locally at the Pasteur Institute in HCMC from the BCG-Pasteur 1173P_2_ strain was used, whereas the BCG-Danish strain produced by the Statens Serum Institute in Denmark was used in Tanzania.^11^ Genetically different BCG vaccine strains could induce different immune responses due to differences in the underlying mechanism and magnitude of the immune response, potentially leading to variations in the protective efficacy against TB,^28^–^30^ and to differences in delayed type hypersensitivity responses, the underlying mechanism for the induration caused by the TST.^31^ A study conducted in Turkey using the same strain as in Vietnam showed a decreased odds for infection with *M. tuberculosis* in BCG-vaccinated children compared to non-vaccinated children^32^ when using IGRA, but not when assessed with TST, unless a larger TST induration size cut-off was applied for the children with a BCG scar (15 mm) than for those without a BCG scar (10 mm). However, using a larger induration size cut-off for children with a BCG scar may bias the association between BCG status and TST positivity as cross-reactivity to NTM could cause false-positive TST results.^18^

### Limitations

Our study had some shortcomings. First, we could not account for potential residual confounding by factors such as socio-economic status and HIV infection. Due to a decreased immune response in HIV infection, TST indurations are more often negative (zero) in HIV-infected individuals with suppressed cellular immunity. However, HIV infection in children was infrequent in Vietnam,^27^ and we do not expect children who are HIV-infected at birth to have been among the survey populations. Second, we relied on the presence of a BCG scar to determine vaccination status, while a scar does not always form after vaccination. This would result in masking any protective effect of BCG vaccination. However, the proportion of children who do not develop a scar after BCG vaccination varies from 0%^33^ to 25%,^34^ limiting the potential of bias in our study. Data on BCG status were collected by several observers, and there may have been inter-observer variability. We dealt with digit preference in induration size measurement by taking 2 mm increments so that a value at a multiple of 5 was always combined with an adjacent value; however, uncontrolled misclassifications may have remained. Finally, unlike longitudinal studies,^8^ the surveys in our study could not take into account reversions and reconversions, as they had a cross-sectional design. This may have resulted in a conservative estimate of the protective efficacy of BCG against *M. tuberculosis* infection.

Current tests for *M. tuberculosis* infection, including TST, indicate prior exposure, not necessarily ongoing infection. Furthermore, the specificity of the TST is affected by false-positive results due to cross-reactivity with NTM and BCG vaccination.^18^,^35^ A TST is usually considered positive (i.e., a person is considered infected) if the induration caused by the skin test is larger than 10 mm.^18^,^35^ Despite its limitations, our study results are important for determining the role of BCG in immunisation programmes, clinical trials and future implementation strategies in high-burden countries.

## CONCLUSION

We found no evidence of a protective effect of BCG vaccination against *M. tuberculosis* infection among Vietnamese schoolchildren as measured by an inverse association of BCG with positive TST. These findings are unexplained and may be due to geographical differences, and possibly, the infecting *M. tuberculosis* genotype.
